# Fasting Versus Non-Fasting Total Testosterone Levels in Women During the Childbearing Period

**DOI:** 10.7759/cureus.35462

**Published:** 2023-02-25

**Authors:** Emad Baqer Ali, Ali Alhamza, Ibraheem A Zaboon, Haider A Alidrisi, Abbas A Mansour

**Affiliations:** 1 Endocrinology, Diabetes and Metabolism, Faiha Specialized Diabetes, Endocrine and Metabolism Center, Basrah, IRQ; 2 Adult Endocrinology, Faiha Specialized Diabetes, Endocrine and Metabolism Center, Basrah, IRQ; 3 Endocrinology, Faiha Specialized Diabetes, Endocrine and Metabolism Center, Basrah, IRQ; 4 Diabetes and Endocrinology, University of Basrah College of Medicine, Basrah, IRQ; 5 Diabetes and Endocrinology, Faiha Specialized Diabetes, Endocrine and Metabolism Center, Basrah, IRQ

**Keywords:** hirsutism, hyperandrogenism, fasting testosterone, testosterone in women, testosterone

## Abstract

Background

Total testosterone in men should be measured in the fasting state early in the morning with at least two samples according to guidelines. For women, no such a recommendation is available despite the importance of testosterone in this demographic. The aim of this study is to evaluate the effect of fasting versus non-fasting state on the total testosterone levels in women during the reproductive period.

Methods

This study was conducted at Faiha Specialized Diabetes, Endocrine and Metabolism Center in Basrah, (Southern Iraq) between January 2022 to November 2022. The total enrolled women were 109; their age was 18-45 years. The presentation was for different complaints; 56 presented for medical consultation with 45 apparently healthy women accompanying the patients as well as eight volunteering female doctors. Testosterone levels were measured by electrochemiluminescence immunoassays using the Roche Cobas e411 platform (Roche Holding, Basel, Switzerland). Two samples were collected from each woman; one was fasting and another was non-fasting the following day, and all samples were taken before 10 am.

Results

For all of the participants, the mean ± SD fasting was significantly higher as compared to the non-fasting testosterone (27.39±18.8 ng/dL and 24.47±18.6 ng/dL respectively, p-value 0.01). The mean fasting testosterone level was also significantly higher in the apparently healthy group, (p-value 0.01). In women who presented with hirsutism, menstrual irregularities and or hair fall, no difference was seen in the testosterone levels between fasting and non-fasting states (p-value 0.4).

Conclusion

In the apparently healthy women of childbearing age, serum testosterone levels were higher in the fasting versus the non-fasting states. In women who presented with complaints of hirsutism, menstrual irregularities, and or hair fall, the serum testosterone levels were not affected by the fasting states.

## Introduction

The total testosterone levels in men should be measured in the fasting state in early in the morning with at least two samples according to guidelines [1,2]. In women no such solid recommendation has been made despite the importance of testosterone; it acts directly as an androgen and is an obligatory precursor for the synthesis of estradiol [3]. Testosterone exerts a physiological impact on reproductive and non-reproductive tissues in women. Hyperandrogenism is a cardinal feature for women with polycystic ovary syndrome (PCOS) and it is agreed to by all to be an important component for diagnosing this condition, in association with oligo-anovulation and/or polycystic ovarian morphology [4]. Rising evidence indicates PCOS with hyperandrogenism may have a more unfavourable metabolic profile than patients with the normal androgenic phenotype, suggesting a potential increment in the risk of cardiovascular disease [5]. The effect of endogenous androgens as a risk for type 2 diabetes is well established in women with documented hyperandrogenism as polycystic ovary syndrome (PCOS) is a condition linked to increased insulin resistance [6]. Sexual function in women is positively associated with the levels of testosterone [7]. During the reproductive period, testosterone is produced by the ovaries and by peripheral conversion of dehydroepiandrosterone (DHEA) and androstenedione; both are pre-androgens synthesized by the adrenal glands and ovaries [8]. About 50% of testosterone is produced by ovaries and the adrenal glands (25% for each), while the peripheral conversion of androgens contributes equally to the rest of the testosterone that circulates in the blood [9]. Testosterone concentration begins to rise in girls, approximately at the age of six to eight years, when the adrenal zona reticularis maturation leads to increased production of DHEA and dehydroepiandrosterone sulfate (DHEA-S) [8], indicating the onset of adrenarche. With the onset of ovulation, cyclical production of testosterone by the ovaries begins when the concentrations peak in mid-cycle and stay high during the luteal phase [10]. Endogenous androgen concentration decreases steadily after the age of 30 years [11]. Women will lose about 60% of their total androgen pool when reaching menopause [8]. Most circulating testosterone is conjugated with proteins; about 66% is bound to sex hormone-binding globulin (SHBG), 30% to albumin and only 2-4% of testosterone remains unbound (free testosterone), the form which is considered to be active. Plasma levels of androgens (testosterone and dihydrotestosterone (DHT) are very low in women, however, they have high androgen receptor affinity and therefore have solid androgenic properties, while the adrenal androgen DHEA-S, despite its high plasma concentrations has a low affinity for androgen receptors, and is considered mainly a precursor androgen [12]. To evaluate excess androgen as well as deficiency, reference ranges based on accurately measured levels of total testosterone are indispensable [7]. The highly sensitive and specific liquid chromatography-tandem mass spectrometry (LC-MS/MS) was evaluated to be superior to the conventional immunoassays for the estimation of the sex hormone at low concentrations, especially in women [13, 14]. However, measurement of testosterone using conventional immunoassays in clinical practice is appropriate if liquid chromatography and tandem mass spectrometry assay are not available, as recommended by Global Consensus Position Statement on the Use of Testosterone Therapy for Women [15]. The normal range of testosterone in women measured by conventional immunoassays during reproductive age is 15-46 ng/dL [16]. Evaluation of testosterone in men may be affected by circadian variation, whereas food intake mildly suppresses testosterone concentrations [17,18]. Various stressors may cause testosterone concentrations to fluctuate; up to 30% of reductions can occur during the acute phase of illness [19]. Furthermore, several medications, like glucocorticoids, may also temporarily affect testosterone secretion. Testosterone concentrations in women vary according to the phase of the menstrual cycle and the body mass index, and this makes establishing normal ranges more cumbersome [20]. For increasing specificity, most societies recommend evaluating androgen excess in women during the follicular phase [21].

The aim of this study is to evaluate the effect of fasting versus non-fasting on the total testosterone levels in women during the reproductive period.

## Materials and methods

Study design, place and time

A cross-sectional study was conducted at the Faiha Specialized Diabetes, Endocrine and Metabolism Center (FDEMC) in Basrah, Southern Iraq, between January 2022 and November 2022.

Participants

In this study, 109 women were recruited, their ages ranging from 18-45 years. Of the total women, 56 presented for different medical consultations (18 of them for hirsutism, 25 with irregular menstrual cycles, 13 with hair fall), 45 apparently healthy women accompanying the patients as well as eight female doctors. Verbal consent was taken from each participant after explaining the aim of the study in accordance with the ethical standards of the FDEMC Research Committee, from which the ethical approval was obtained (ref #60/33/21), and with the 1964 Declaration of Helsinki and its later amendments or comparable ethical standards. The inclusion criteria included female gender and being between 18-45 years of age and the exclusion criteria were being below 18 years and above 45 years of age, pregnant women, women on oral contraceptive pills or other hormonal therapy, on antiandrogenic drugs, prolactinoma, pituitary surgery, or women with hypo- or hypergonadotrophic hypogonadism, premature ovarian insufficiency or history of oophorectomy, as well as those who did not attend the second appointment on the following day. Every woman was evaluated for measurement of height (meter), and weight (kilogram) from which the body mass index (BMI) was calculated.

Biochemical analysis

A random sample of venous blood is taken during fasting or not fasting to measure the level of total testosterone and other related hormones, as required to diagnose the diseased condition of the woman; then a second appointment to measure testosterone only is set in a reverse manner regarding fasting state. A sample of 5 ml of venous blood was taken at each appointment for the time 9:00-10:00 am and was labelled as fasting or non-fasting, accordingly. The above samples were not specifically collected for the study and were taken as part of a routine evaluation and management of the included patients. Total testosterone was measured from separated serum by electrochemiluminescence immunoassays (ECLIA), Roche® Cobas e411 platform (Roche Holding, Basel, Switzerland). The normal reference range of testosterone in women in this study was 15-45 ng/dL (coefficient variance <6%) [16].

Statistical analysis

All data were analyzed using the statistical package of the social science program (SPSS v. 26, IBM Corp., Armonk, NY). Mean was used as an expression of numerical data and the percentage was used to express nominal data. A two-sided paired T-test used for comparing means with (p-value <0.05) was considered to be significant.

## Results

The means of age, body mass index (BMI), luteinizing hormone (LH), follicular stimulating hormone (FSH) and estradiol, with frequency (percentage) of marital status, complaints and diabetic status of the total number of 109 participants are presented in Table 1. Of these, 69 (63.4%) were married and 40 (36.6%) were unmarried. The mean age was 28.46±8.02 years, BMI 29.2±6.9 kg/m2, LH 6.88±5.69 mIU/mL, FSH 5.84±3.87 mIU/mL and estradiol 88.78±61.86 pg/dl. Twenty women had diabetes (18.3%). Participants who were apparently healthy were 53 (48.6%), and the other 56 (51.4%) complained of hirsutism (18; 16.5%), irregular menstrual cycle (25; 22.9%) and hair fall (13; 11.9%).

**Table 1:** General characteristics of participants (N=109) Abbreviations: DM, diabetes mellitus; BMI, body mass index; LH, luteinizing hormone, FSH, follicular stimulating hormone, SD, standard deviation, N, number of participants

General characteristics of participants(N=109)		
Age (years)	Mean ± SD	28.46±8.02
Marital status, N (%)	Married	69(63.4)
	Unmarried	40(36.6)
Complaints, N (%)	Hirsutism	18(16.5)
	Irregular cycle	25(22.9)
	Hair fall	13(11.9)
	Apparently healthy	53(48.6)
DM, N (%)	Yes	20(18.3)
	No	89(81.7)
BMI, (kg/m2)	Normal weight (%)	27(24.8)
	Overweight (%)	38(34.9)
	Obese (%)	44(40.4)
LH (mIU/mL)	Mean±SD	6.88±5.69
FSH (mIU/mL)	Mean±SD	5.84±3.87
Estradiol (pg/dL)	Mean±SD	88.78±61.86

For all of the participants, the mean±SD fasting was significantly higher as compared to the non-fasting testosterone (27.39±18.8 ng/dL and 24.47±18.6 ng/dL respectively, p-value 0.01) as shown in Table 2. The mean fasting testosterone level was also significantly higher in the apparently healthy group, (p-value 0.01). In women presenting with hirsutism, menstrual irregularities and or hair fall, no difference was seen in the testosterone levels between fasting and non-fasting states (p-value 0.4). 

**Table 2:** Comparison between fasting and non-fasting testosterone in the study groups Abbreviation: SD, standard deviation

Group	Fasting testosterone (mean ± SD)	Non-fasting testosterone (mean ± SD)	Mean difference (mean ±SD)	P value
All participants (N 109)	27.39 ± 18.84	24.46 ± 18.62	2.92 ± 12.36	0.01
Apparently healthy (N 53)	25.0 ± 15.82	20.38 ± 15.17	4.61 ± 12.59	0.01
Patients with hirsutism, irregular menstrual cycle, and or hair fall (56)	29.65 ± 21.20	28.33 ± 20.78	1.32 ± 12.04	0.4

To study the effect of BMI on fasting and non-fasting testosterone levels, the BMI was subdivided into three groups (18-24.9kg/m2, 25-30kg/m2 and >30kg/m2). The mean ± SD of fasting and non-fasting testosterone was 27.39±18.8 and 24.47±18.6 respectively. Women with obesity tend to have higher mean testosterone levels as compared to other BMI groups both in fasting and non-fasting state. However, these differences were not significant. The fasting testosterone level was higher independent of BMI categories (Table 3).

**Table 3:** The effect of the three groups' BMI on the fasting and no-fasting total testosterone levels Abbreviations: BMI, body mass index, SD, standard deviation

BMI	Fasting testosterone		Non-fasting testosterone	
	Number	Mean±SD	Number	Mean±SD
Normal weight	27	26.63± 18.87	27	22.24± 13.98
Overweight	38	25.20± 18.06	38	22.62± 16.18
Obese	44	29.74± 19.63	44	27.42± 22.64
Total	109	27.392±18.8	109	24.469±18.6
p-value	0.243		0.445	

## Discussion

The decision to measure total testosterone in women in the state of fasting or not is important from the clinical point of view as food may affect testosterone levels. This was clearly stated by guidelines in men [1,2] but not in women. Despite the evidence that testosterone and other androgens are essential for reproductive function and health [22], there are a lot of obstacles to explaining the physiology of androgen in females. There were difficulties in accurately measuring low levels of testosterone in women; finding a study comparing and investigating the effect of fasting states on testosterone measurement between men and women was also difficult.

This study showed that in the apparently healthy women, fasting testosterone was higher than non-fasting testosterone but not in women presented with hirsutism, menstrual irregularities, and or hair fall. We found fasting would increase total testosterone in women by around 9%. Compared to male studies, Tremellen et al. found that eating fast food, mixed meal or high fat produced a 25% reduction in serum total testosterone during the first hour of eating, with levels continuing to be suppressed below the fasting level for about four hours, without significant effect on the levels of gonadotropin; however, consuming intravenous fat had no effect on testosterone levels, suggesting that the mechanism may be operated through the gastrointestinal tract that provokes an indirectly mediated response [23]. A decrease in circulating testosterone and free testosterone after a fat-containing meal was also described by Meikle et al [24]. In another study, Gagliano-Jucá et al. showed that testosterone reduced after oral ingestion of 75 g glucose, the mean drop had a nadir of 100.8 ng/dl, but levels appeared to be reactive and returning towards baseline within 120 min [25]. Similarly, Caronia et al. [26] also showed a reduction of 25% in the level of testosterone following an oral glucose load; however, there was no change in cortisol, LH or SHBG levels. Food intake induces the release of gastro-pancreatic hormones that modulate splanchnic blood flow with the modulation of an affinity of testosterone to binding proteins. This lead to increased testosterone translocation to the peripheral tissues with higher plasma clearance. Another possible mechanism to explain is the presence of specific factors in food that may enhance the intracellular testosterone uptake directly or by testosterone complex formation, which is translocated into the cells [27]. These effects of meal intake in male studies appeared comparable to our study in the apparently healthy female group and not in the hyperandrogenism group. This finding may be explained by the differences between men and women in the testosterone level, mechanisms and major organs of secretion, compositions of the androgens, and the pathophysiology of female hyperandrogenism. 

A study comparing the change in testosterone levels as an effect of different food constituents in women with PCOS which was conducted by Katcher et al., showed a reduction in testosterone levels of 27% within two hours after ingestion of a Western meal, or a low-fat, high-fat and high-fibre meal in women with PCOS. However, testosterone levels continue to drop for two hours more after the Western meal and are high-fat compared with the high-fibre and low-fat meal, indicating that the postprandial testosterone is affected by the meal composition independent of caloric load [28]. But in the presented study, no difference was seen in the testosterone level as a result of the fasting or non-fasting state in women complaining of hirsutism, menstrual irregularities, and or hair fall. In this study, random non-fasting samples were taken after the usual regular breakfast and not after a high-fat meal like in the previous study. 

The effect of BMI with fasting and non-fasting testosterone level showed no significant difference in this study, consistent with Panidis et al. [29] that reported that serum testosterone levels changes occur similarly after oral administration of 75 g dextrose to the normal weight, overweight and obese women, as a result of Parra et al [30].

This study has some limitations. First, the sample size was small. Second, the unavailability of gold standard testosterone measurement technique by LC/MS. Third, data from other ethnicities should form part of future studies.

## Conclusions

In the apparently healthy women of childbearing age, serum testosterone levels were higher in the fasting versus the non-fasting states. In women who presented complaining of hirsutism, menstrual irregularities, and or hair fall, the serum testosterone levels were not affected by the fasting states.
